# Building clinical trial priorities at the University of Rwanda

**DOI:** 10.1186/1745-6215-15-467

**Published:** 2014-11-27

**Authors:** Jeanine Condo, Brenda Kateera, Eugene Mutimura, Francine Birungi, Albert Ndagijimana, Stefan Jansen, Julius Kamwesiga, Jamie I Forrest, Edward J Mills, Agnes Binagwaho

**Affiliations:** School of Public Health, College of Medicine & Health Sciences, University of Rwanda, Kigali, Rwanda; Institute of HIV Disease Prevention and Control, Rwanda Biomedical Centre, Kigali, Rwanda; Global Evaluative Sciences, Vancouver, Canada; Ministry of Health, Kigali, Rwanda

**Keywords:** commentary, randomized clinical trials, Rwanda, health systems, research capacity

## Abstract

After the genocide in Rwanda, the country’s healthcare system collapsed. Remarkable gains have since been made by the state to provide greater clinical service capacity and expand health policies that are grounded on locally relevant evidence. This commentary explores the challenges faced by Rwanda in building an infrastructure for clinical trials. Through local examples, we discuss how a clinical trial infrastructure can be constructed by (1) building educational capacity; (2) encouraging the testing of relevant interventions using appropriate and cost-effective designs; and, (3) promoting ethical and regulatory standards. The future is bright for clinical research in Rwanda and with a renewed appetite for locally generated evidence it is necessary that we discuss the challenges and opportunities in drawing up a clinical trials agenda.

## Background

The depletion of the health system in Rwanda after the 1994 genocide resulted in a paucity of health workers capable of providing clinical services across the country. As a result, the priorities for initially rebuilding the health system have focused on the training of clinicians and cadres of health service workers, and on supplying humanitarian aid. More recently, Rwanda has reached a stabilization period in providing clinical service capacity and is now expanding priorities to build policies that are based on locally relevant evidence [1].

In 2014, priorities for building research infrastructure include the institutionalization of research capacity through human resource development, investments in program evaluation, and educational assistance [2, 3]. Determining how future research priorities are developed means making difficult decisions about where to make research investments and how to prioritize efforts in research. Should these investments be in basic sciences, clinical research or operational evaluations? In the context of limited resources, there is a consensus that Rwanda should focus on an evidence-based research agenda to optimize decision-making. This will inevitably require that we build a robust infrastructure for clinical trials to inform the scale-up of indigenous research for the most effective interventions. Herein we list the three pressing challenges related to building a clinical trial infrastructure: (1) building educational capacity; (2) encouraging the development of relevant and novel interventions to test; and, (3) promoting ethical and regulatory standards.

### Building educational capacity

A first step in building clinical trial capacity is providing educational opportunities for Rwandans and our global partners. While Rwanda has importantly invested in clinical staff, focusing on physicians and other grades of front-line health workers, it has invested much less in the development of statistical and methodological health professionals [3, 4]. The most senior health staff in Rwanda are typically the first cohort of physicians, nurses and other professionals trained after the genocide. These individuals already hold senior positions within government and health facilities and have historically worked in an environment where training and orientation has focused on improving the efficiency and quality of clinical service delivery. Although a Master of Public Health program and a new Doctor of Philosophy program are available through universities in Rwanda, no specific and targeted educational opportunities related to clinical trials exist [4]. A focus on recruiting faculty with experience in instructing a course in clinical trial design and analysis will further improve the training of both masters and doctoral level public health students. Efforts to rapidly build clinical trial capacity include partnership with major universities in the global north, mentorship, training workshops, and attracting experienced Rwandan faculty; Rwandans currently working in the university but also Rwandans outside of the university including those from the diaspora. There is recognition within the University of Rwanda and the Ministry of Health that long-term mentoring between experienced researchers and new investigators is needed to achieve not only basic knowledge of clinical trial methods but also to assist in problem solving when trials go awry. Examples are current mentorships between the Rwanda Biomedical Centre and Stanford University for HIV trials, a collaboration between the Rwanda Biomedical Centre and the University of Ottawa for a dietary supplement trial [5], and ongoing collaborations between the Ministry of Health and Emory University via the Project San Francisco. These have primarily focused on HIV projects and will now need to expand to other disease areas.

### Encouraging relevant interventions

It is crucial that we prioritize the testing of relevant and novel interventions that are in accordance with the burden of disease and needs of the country. While standard pairwise randomized trials of drugs versus placebos are unlikely to be among the first trial designs implemented (owing to their complexity in design and high cost burden), there is a need for innovative designs to answer locally relevant questions that compare interventions beyond simply drug-placebo interactions. For instance, currently in Rwanda there is a need to evaluate the effectiveness of many innovative programmatic interventions [6, 7]. Examples of locally relevant clinical trials within Rwanda have included the ‘Pay for Performance’ economic intervention for the improvement of the health system [8]; educational interventions for health behavior change; and task shifting for voluntary medical male circumcision [9]. As we further develop capacity for clinical trials, there may be a focus on different study designs, including cluster designs, implementation science strategies, such as stepped-wedge designs, and adaptive clinical trials that allow for protocol changes according to the needs of the local population and government [10]. Medical guidelines, for example, HIV treatment guidelines, change rapidly, and trials designed to be locally relevant need to adapt to guideline changes. For example, the Strategic Timing of Antiretroviral Treatment trial [11], a multinational trial evaluating at what CD4 cell count to start antiretroviral therapy (≤350 cells mm^3^ versus immediate therapy), would not be currently relevant in Rwanda, as guidelines have moved from ≤350 in 2008 to ≤500 in 2014 and are likely to move to immediate treatment at diagnosis by 2015. Furthermore, as Rwanda is experiencing an epidemiological transition away from infectious diseases, prioritizing of interventions that address diseases not traditionally deemed of importance to the region will be increasingly necessary. These include non-communicable diseases, mental health concerns, and rehabilitation disorders. Treating non-communicable disease is now a national priority, but what conditions of non-communicable disease should be prioritized is unclear [1, 12]. For example, simplified cancer therapies might work for some cancers and not for others [13]. Preventive strategies for hypertension will probably yield a greater return on investment than prioritizing treatment of hypertension and its associated morbidities [14]. Strategies to improve mental health are probably unique in Rwanda, as the genocide has resulted in mass mental trauma. What types of evidence from other settings can be utilized in this setting? Should rehabilitation interventions target the physical or neurological disorders that resulted from the genocide? These questions illustrate how priorities have changed from the hitherto expected tropical diseases to conditions that are longer term and may require lifelong assistance.

### Promoting ethical standards

Lastly, there is a need for an ethical and regulatory framework for building clinical trial infrastructure. A major concern [9] when determining what types of clinical trial to embark upon is who sets the agenda of what interventions should be assessed? There are arguably five major players interested in conducting clinical trials in Rwanda: the Rwandan government; academics and their foreign funders (for example, the US National Institutes of Health); non-government organizations; and the pharmaceutical industry. While these are the major actors interested in clinical trials, the financial sponsors of clinical trials may have a greater say in what interventions are assessed than the representative actors. The Rwandan government and individual academics rarely sponsor clinical trials, owing to limited access to funds or the restricted mechanisms of accessing available funding opportunities. It is the responsibility of both Rwandan academics and their collaborators to ensure that the results of a trial are locally contextualized and support the research agenda set by local researchers and government officials, to inform policy and decision makers. Thus far, only trials related to HIV (vaccines, microbicide and male circumcision) have been implemented in the country, through partnerships with industry to provide financial support and products. Similarly, in most academic partnerships, academics from the West may have opportunities to procure more funding than their Rwandan counterparts, and so partnerships may not be always equal. There is a clear need for ethical and regulatory boards in Rwanda to determine whether the clinical trials proposed investigate locally relevant questions that will benefit the population, in addition to determining issues of design and integrity. In some cases, protocols of clinical trials presented to institutional review boards are not systematically followed up with respected to ethical integrity. For example, the Rwandan institutional board dealing with ethics and human subjects does not have a data safety and monitoring committee for clinical trials in-country to oversee its regulations and trials often need to commission other boards for these services. These are systems that must be strengthened alongside the growth of educational capacity and the stimulation of novel interventions, if we are to better address indigenous health needs in Rwanda.

## Conclusions

The future is bright for clinical research in Rwanda, but immediate challenges remain. A great deal of work has already been done in improving the healthcare system and there is an enormous desire for the evaluation of locally relevant research. However, achieving an environment that can conduct high-quality research will require both internal expertise in the country and strong partnerships with external experts, as well as opportunities for growth of local investigators, and the formation of policies that encourage research while avoiding exploitation. Going forward, building research infrastructure and capacity will be a key pillar in the development of a stronger Rwanda.
